# Cadonilimab plus anlotinib effectively relieve rare cardiac angiosarcoma with multiple metastases: a case report and literature review

**DOI:** 10.1007/s00392-023-02251-y

**Published:** 2023-07-05

**Authors:** Ziyue Zeng, Zijie Mei, Min Chen, Hong Cao, Qingming Xiang, Huanhuan Cai, Zhibing Lu, Hui Qiu

**Affiliations:** 1 Department of Cardiology, Zhongnan Hospital of Wuhan University, No. 169 Donghu Road, Wuchang District, Wuhan, 430071 China; 2 Department of Gynecological Tumor Radiotherapy and Chemotherapy, Hubei Key Laboratory of Tumor Biological Behaviors, Hubei Cancer Clinical Study Center, Zhongnan Hospital of Wuhan University, No. 169 Donghu Road, Wuchang District, Wuhan, 430071 China; 3 Department of Pathology, Zhongnan Hospital of Wuhan University, Wuhan, Hubei China

Sirs,

Cardiac angiosarcoma (CA) is an uncommon primary tumor that originates from subendothelial pluripotent cells within the intimal wall of blood vessels [1]. Primary CA is a rare diagnosis, with sarcomas accounting for approximately one-third of all primary cardiac tumors (0.017–0.02% of all cardiac tumors). Among malignant sarcomas, 30–50% are angiosarcomas. Despite significant advancements in imaging technology, rare intracardiac tumors remain challenging to identify and evaluate during early stages. Consequently, histological and non-invasive imaging methods are still required for accurate diagnosis [2]. To date, three primary cancer treatments have been developed: surgery, chemotherapy, and radiotherapy. These treatments can cure early-stage cancers but often prove ineffective for advanced or recurrent cancers [3]. For CA with multiple metastases, comprehensive systemic therapy may be considered as the main treatment alongside surgery. However, the heart's sensitivity to radiation damage, which can lead to cardiomyopathy and chronic pericarditis, renders radiotherapy unsuitable for cardiac sarcomas. Furthermore, the cardiotoxicity of anthracycline drugs, typically the first choice for chemotherapy in sarcomas, may limit their use [4]. Cancer immunotherapies have emerged as a potential fourth cancer treatment [5].

Sarcomas are rare, representing approximately 1% of all cancers in adults and 15% in children. Metastases are often present at the time of diagnosis [6]. Treatment options for metastatic sarcomas are limited. Besides systemic chemotherapy, various immune-based treatments, such as immune checkpoint inhibitors (ICIs), therapeutic vaccines, and adoptive cell therapy, have been explored for sarcomas. Single-agent immunotherapy has proven effective for certain sarcoma subtypes, while combined immunotherapy appears to yield better response rates [7]. Angiosarcoma (AS) is a rare and highly invasive sarcoma, accounting for less than 1% of all sarcomas [8]. AS can develop throughout the body, with the most common sites being skin lesions, particularly in the head and neck, as well as soft tissue, internal organs, bones, and the retroperitoneum [9]. The treatment of AS presents its own challenges. Conventional treatment includes radical surgery to achieve the best surgical margin [10]. However, in metastatic cases, only cytotoxic chemotherapy, targeted therapy, and more recently, immunotherapy can be considered. At present, targeted therapy, antiangiogenic drugs, and immunotherapy are being investigated as promising treatments for angiosarcoma [11, 12]. The emergence of cancer immunotherapies as a potential fourth cancer treatment further underscores the importance of exploring these novel approaches to address the limitations of existing treatments [13]. As the understanding of tumor biology and the immune system advances, it is anticipated that these innovative therapies may offer new hope for patients with AS and other rare cancers.

In this report, we present a case detailing the diagnosis, treatment, and outcome, suggesting that the combination of cadonilimab and anlotinib may be a potential treatment option for CA. Further studies are necessary to confirm the benefits of this regimen in patients with angiosarcoma.

The patient, a 52-year-old female, was admitted to the Department of Oncology and Department of Radiation Oncology at Zhongnan Hospital of Wuhan University (Wuhan, China) on June 13, 2022. Prior to this, she had been hospitalized in the structural heart disease ward of the same hospital for half a month starting February 15, 2022, due to intermittent coughing, sputum production, and decreased physical strength. Transthoracic echocardiography revealed a 4.8 × 6.7 cm left atrial mass. Cardiac MRI and CT angiography further indicated a pedicled mass connected to the left atrial lateral wall, projecting toward the left atrial appendage and ventricle (Fig. 1). Due to the ultrasound findings showing a significant left atrial mass, reduced left atrial space, and proximity to the mitral valve with a high risk of shedding, atrial tumor resection and tricuspid valvuloplasty were performed under hypothermic cardiopulmonary bypass and general anesthesia on February 17. Histopathological examination revealed a highly malignant sarcoma. Combined with microscopic morphology, tumor location, and immunophenotype, angiosarcoma was considered (Fig. 2).

**Fig. 1:**
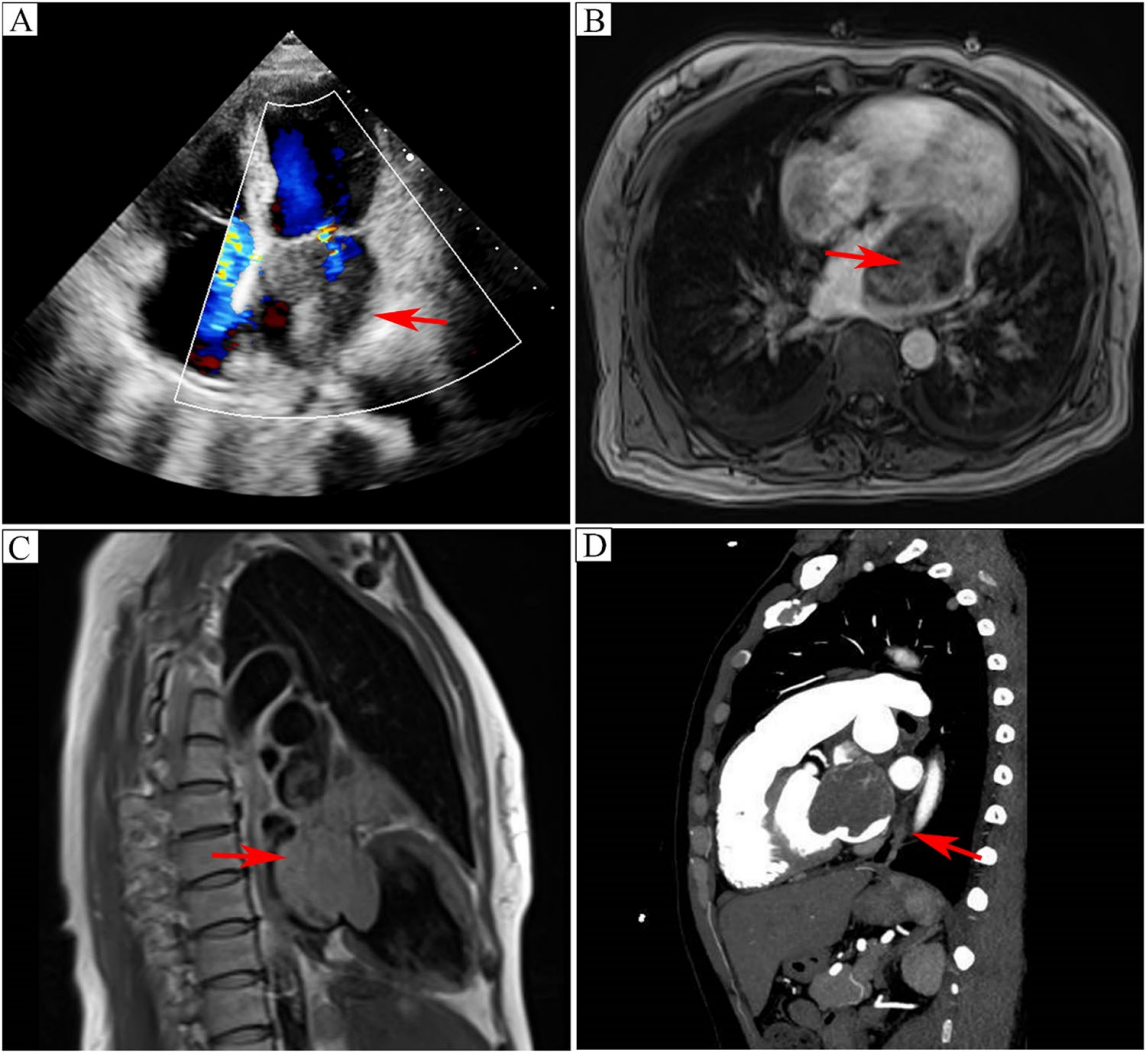
Transthoracic echocardiography, cardiac MRI, and CT angiography.** A** Transthoracic echocardiography captured the left atrial giant sarcoma, exhibiting a slightly weak echo and measuring 4.8 × 6.7 cm. **B**, **C** Transverse and sagittal cardiac MRI revealed a protruding and compressive left atrial mass. **D** Sagittal cardiac angiography demonstrated an irregular mass within the left atrium, locally extending into the left atrial appendage, oscillating in accordance with the cardiac cycle, displaying high activity and notable enhancement

**Fig. 2:**
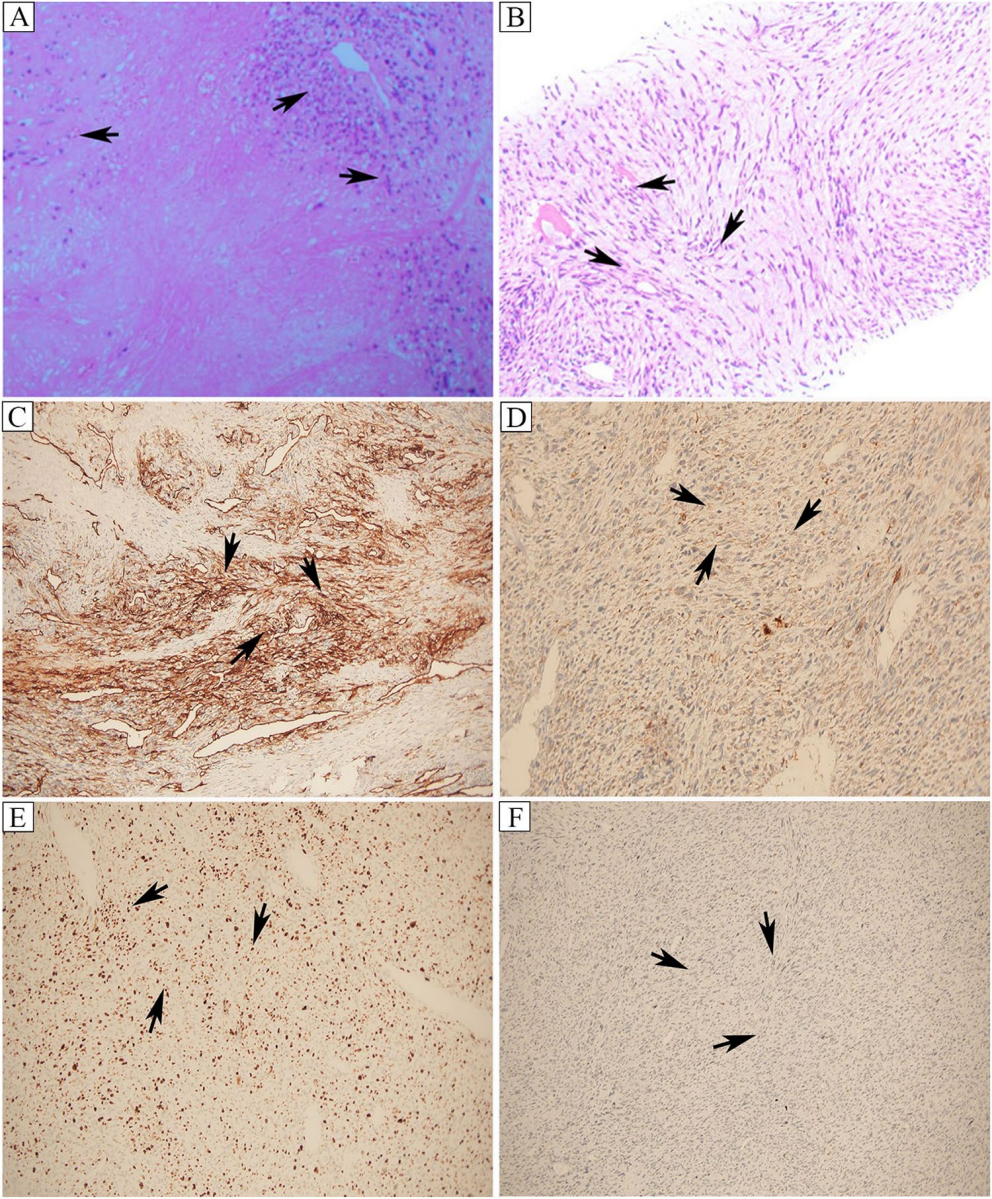
Hematoxylin–eosin and immunohistochemical staining of atrial and left lower limb masses. **A**, **B** Hematoxylin–eosin staining of atrial and left lower limb masses. **C**–**F** Immunohistochemical staining of the atrial mass revealed positive CD34 (+), focally positive CK (focal +), Ki-67 proliferation index (LI: 40%), partially positive MDM2 (partial +), while other markers were negative [Desmin (−), EMA (−), S-100 (−), CD31 (−), ERG (−), SOX-10 (−), etc.]

The patient had no notable family history. In terms of past medical history, the patient was diagnosed with cervical cancer in 2014 and received surgery, radiotherapy, and chemotherapy at the same hospital. The cervical cancer is stage IIB. During concurrent chemoradiotherapy, nedaplatin was used concurrently. After surgery, one cycle of chemotherapy with docetaxel and nedaplatin was administered as a supplement. The patient had no other significant previous medical or surgical history.

After 2 months, multiple metastatic lesions were identified in areas such as the right levator ani muscle, left lung, and left lower limb (Figs. 3, 4). A puncture biopsy of the left lower limb metastasis confirmed metastatic angiosarcoma (Fig. 2). The patient refused chemotherapy, and next-generation sequencing (NGS) of peripheral blood, surgical resection tissue, and femoral soft tissue angiosarcoma show slight variations. There are 14 gene mutations identified in the blood sample, 29 gene mutations in the atrial tumor tissue, and 35 gene mutations in the femur tumor tissue. Microsatellite analysis of both the atrial and femur biopsy tissues indicates MSI-H. Additionally, all three samples (blood, atrial tumor tissue, and femur biopsy tissue) suggest TMB-H. We detected several genomic changes known to be associated with cancer and identified 35 gene mutations, with the first 4 having the highest mutation abundance: CDK12 (c.1047-1G>A), EZH2 (c.893G>A), EPHA3 (c.1095del), and MLH1 (c.1489dup), among others. Functional enrichment analysis of the mutant genes suggested close relationships with mesenchyme development and other processes (Fig. 5). Subsequently, the patient was treated with sintilimab (PD-1 antibody) (200 mg) combined with anlotinib (12 mg) in June 2022. After two treatment cycles, the curative effect was evaluated as progressive disease (iuPD) according to the irRECIST criteria. The patient complained of increased abdominal and thigh pain and difficulty walking during this stage of treatment, which may be more indicative of true progression. Based on NGS results, the patient was expected to respond well to immunotherapy. Consequently, the treatment regimen was modified to cadonilimab (PD-1/CTLA-4 bi-specific antibody) (375 mg) combined with anlotinib (12 mg). After eight treatment cycles, all metastatic foci showed continuous reduction (Figs. 3, 4), and the patient's symptoms significantly improved. The patient is currently continuing treatment under this regimen, tolerating the combination treatment well, and experiencing no severe adverse events (AEs).

**Fig. 3:**
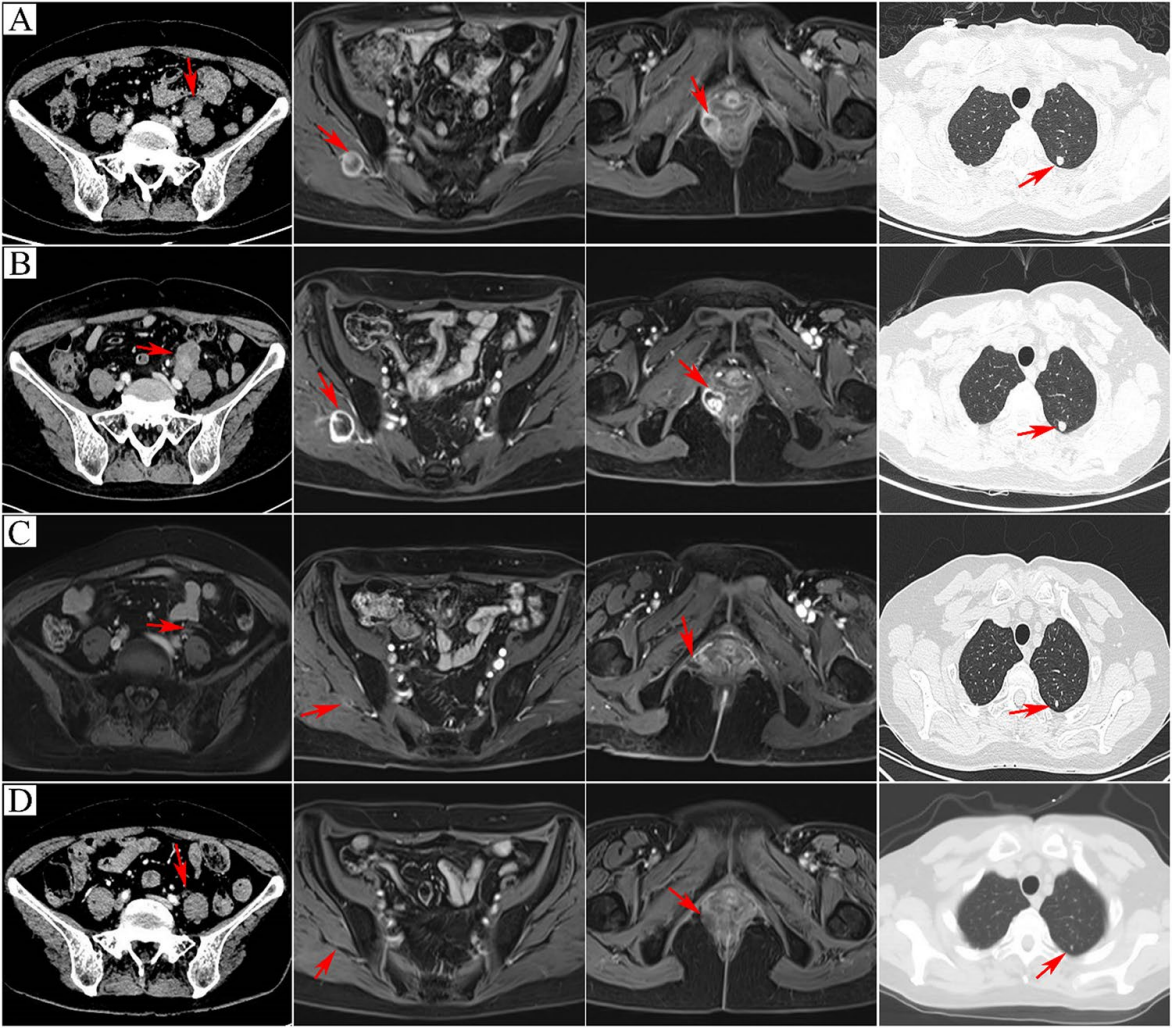
Abdominal MRI and lung CT scans. **A** Postoperative imaging revealed metastatic foci in para-iliac vessels, right gluteus maximus space nodule, right levator ani muscle, and left lung without any drug intervention. **B** After two cycles of sintilimab combined with anlotinib, the metastatic tumor either progressed or remained stable. **C** Following five cycles of cadonilimab combined with anlotinib, the metastatic tumor of the metastatic focus was significantly smaller and improved compared to previous scans. **D** After seven cycles of treatment with cadonilimab combined with anlotinib, the metastatic tumor essentially returned to normal compared to the pre-treatment state

**Fig. 4:**
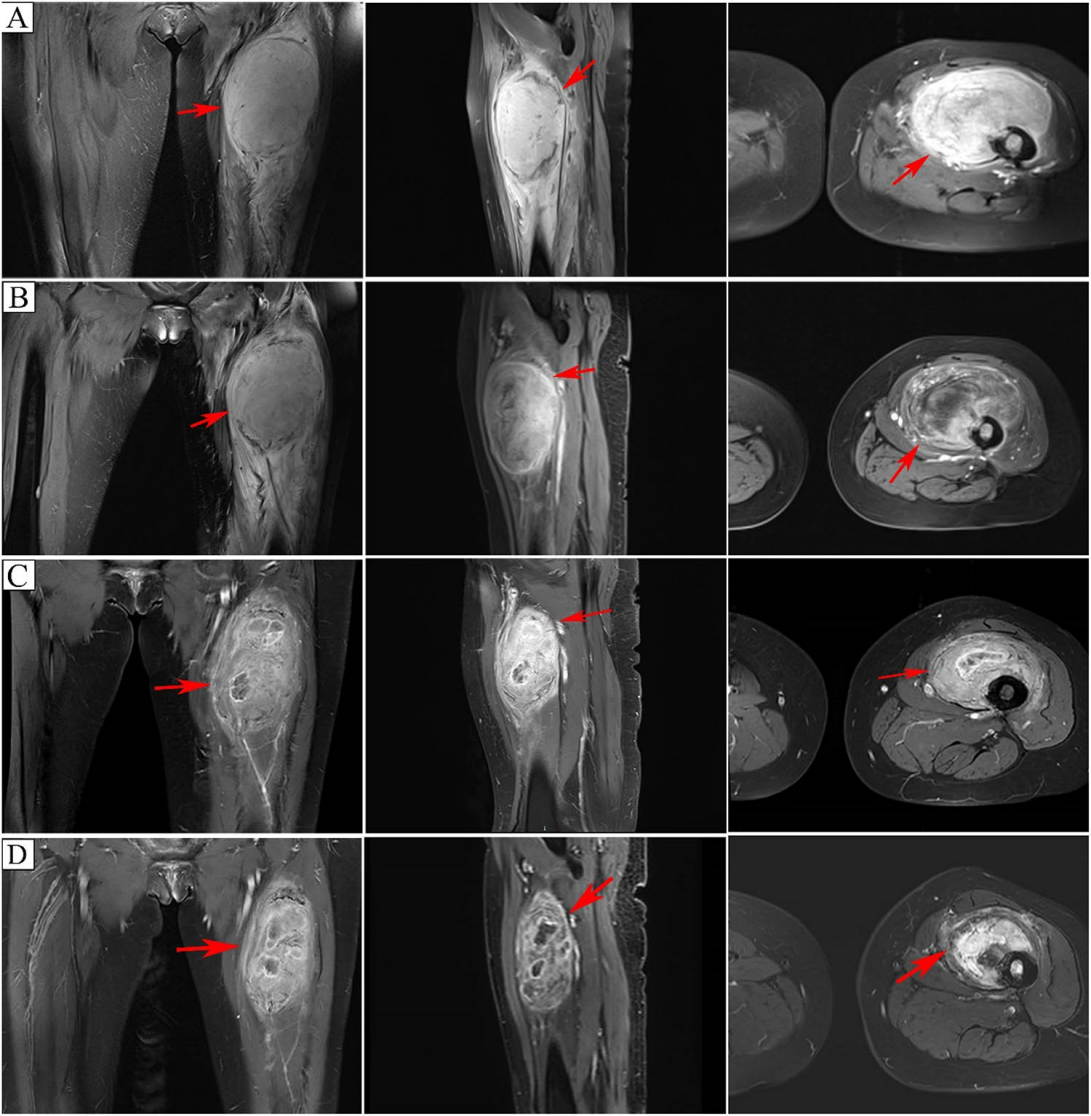
Lower limb MRI. **A** Pre-treatment(cadonilimab plus anlotinib), increased fat-suppressed signal in the upper left femur bone marrow, adjacent large mass (108 × 84 × 71 mm) with high signal intensity, distinct boundary, increased peripheral muscle signal, uneven enhancement, and anterior femoral cortex destruction. **B** Post-treatment, high signal mass in the upper thigh measured 100 × 85 × 61 mm. Anterior left thigh soft tissue mass decreased slightly; other results remained relatively unchanged. **C** After five treatment cycles, high signal mass in the upper thigh measured 92 × 76 × 53 mm. Compared to pre-treatment, the lesion diminished, with multiple central necrotic areas. **D** After seven treatment cycles, high signal mass in the upper thigh reduced to 78 × 66 × 45 mm

**Fig. 5:**
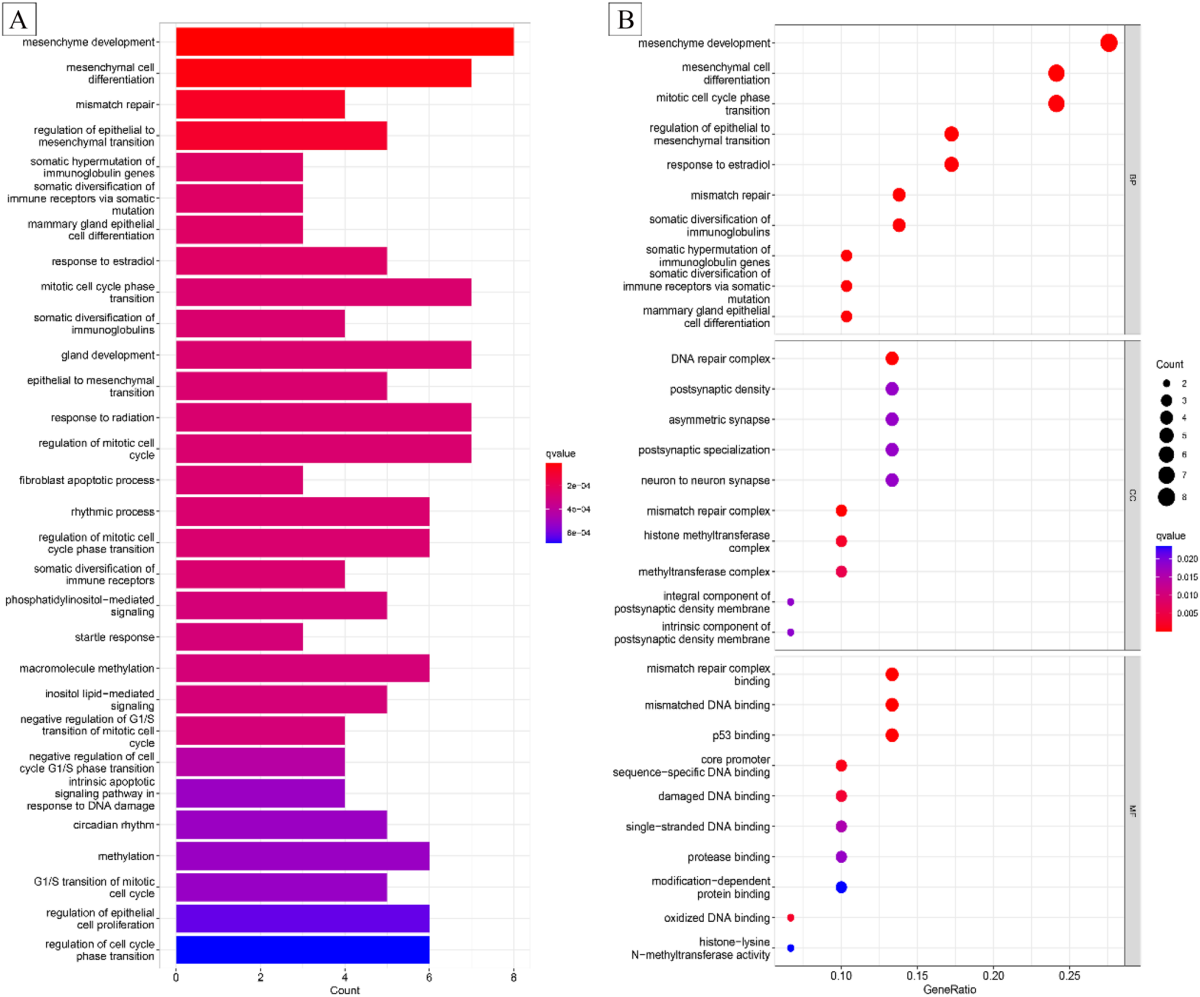
Enrichment analysis of 35 tumor-specific mutant genes in peripheral blood, cardiac angiosarcoma, and femoral soft tissue angiosarcoma. **A** Top 20 Kyoto Encyclopedia of Genes and Genomes (KEGG) pathway enrichment analysis bar chart; **B** top 10 Gene Ontology (GO) enrichment analysis, encompassing biological process, cellular component, and molecular function. The results indicate a strong association with mesenchyme development, mesenchymal cell differentiation, mismatch repair, regulation of epithelial-to-mesenchymal transition, DNA repair complex, mismatch repair complex, mismatched DNA binding, and other related processes

Here, we describe a 52-year-old woman with left atrial angiosarcoma with multiple metastases throughout the body. The disease was significantly controlled upon switching from sintilimab combined with anlotinib to cadonilimab combined with anlotinib.

Sarcoma, a rare and challenging group of over 100 mesenchymal malignant tumor subtypes, often presents with micrometastasis, leading to a 5-year relative survival rate of 16% for metastatic patients [14]. Angiosarcoma (AS), which includes aortic intimal sarcoma, pulmonary artery sarcoma, and atrial arterial intimal sarcoma, is frequently misdiagnosed as protruding atherosclerotic disease, pulmonary thromboembolism, or myxoma. Accurate diagnosis is crucial for timely and effective treatment [15]. AS constitutes approximately 2–3% of all soft tissue sarcomas and is characterized by clinical invasiveness and poor prognosis [16]. While multimodal imaging aids in diagnosis, pathological examination remains the gold standard [17, 18].

Treatment options for AS include surgery, radiotherapy, and chemotherapy. Surgical resection is the preferred method for primary cardiac malignant tumors, with the extent of resection depending on adjacent structures and cardiac cavity involvement [19, 20]. In patients with metastasis, simple palliative resection results in a median survival time of only 6–7 months [21]. No unified standard for AS chemotherapy currently exists, but research suggests that cytotoxic drugs such as platinum, doxorubicin, paclitaxel, and methotrexate may benefit patients not eligible for radical surgery [22]. Advancements in oncology and immunology have led to the emergence of tumor immunotherapy as an anti-tumor approach, complementing surgery, radiotherapy, and chemotherapy [23]. A study analyzing 106 cutaneous AS cases found a correlation between PD-1/PD-L1 expression and AS progression, suggesting that anti-PD-1 antibody therapy could be a potential novel treatment [24]. Targeted drugs and immunotherapy are also being explored as promising treatments for AS [25]. However, immunotherapy for AS requires further research on subtype and/or biomarker specificity. Novel techniques can help discover new immune biomarker relationships and provide a comprehensive understanding of the sarcoma microenvironment through multiplex, spatially resolved RNA, and protein analysis [26]. Studies have shown that a subset of AS is characterized by a high tumor mutation burden (TMB), suggesting that they may respond to immune checkpoint inhibitors (ICIs). Published case reports and small series studies have shown that the initial clinical response of AS patients to ICIs is encouraging [27, 28]. In a phase II clinical trial, the objective response rate (ORR) of ipilimumab combined with nivolumab (dual anti-CTLA-4 and anti-PD-1 blockade) in the treatment of metastatic or unresectable AS was reported to be 25% [29]. Nivolumab combined with ipilimumab demonstrated encouraging ORRs (16%) in patients with sarcoma, including AS, myxofibrosarcoma, and alveolar soft sarcoma, with three AS patients in this study showing remission in the combined regimen group [28].

Therefore, combination therapy is a more recommended method in the clinical study of soft tissue sarcoma. A retrospective study involving 61 patients with advanced soft tissue sarcomas demonstrated improved objective response (OR) values when ICIs were combined with tyrosine kinase inhibitors (TKIs) [30]. Additionally, a study combining Bempegaldesleukin (BEMPEG), a CD122-preferential interleukin-2 pathway agonist, with nivolumab in refractory sarcoma patients yielded the highest ORRs in angiosarcoma and undifferentiated pleomorphic sarcoma [31]. However, ICIs monotherapy has not demonstrated convincing clinical efficacy. Instead, the combination of dual checkpoint inhibitors or other drugs achieved more promising outcomes in treating dedifferentiated liposarcoma, undifferentiated pleomorphic sarcoma, and alveolar soft tissue sarcoma [32]. Anlotinib, a multi-target TKIs, exhibits encouraging clinical activity in lung cancer and sarcoma. A retrospective analysis showed that anlotinib combination therapy is more effective, safer, and long lasting for some advanced sarcomas [33]. Anlotinib combined with anti-PD-L1 therapy displayed potential anti-tumor effects by promoting immune killer cell induction and activation [34]. Research suggests that anlotinib may replace glucocorticoids in combination with anti-PD-1/PDL-1 immunotherapy for brain edema resulting from non-small cell lung cancer brain metastases [35]. A phase II study reported that TQB2450 (a PD-L1 inhibitor) combined with anlotinib produced encouraging ORRs in patients with locally advanced or metastatic soft tissue sarcomas and alveolar soft tissue sarcomas [36], indicating the effectiveness of anlotinib and anti-PD-L1 therapy in treating sarcoma.

Cadonilimab, a PD-1/CTLA-4 bi-specific antibody developed by Akeso, Inc., was approved in China in June 2022 for the treatment of recurrent or metastatic cervical cancer (r/mCC) that showed progression during or after platinum-based chemotherapy [7]. In the cases of cardiac angiosarcoma (AS) documented here, the combination therapy of cadonilimab and anlotinib produced favorable results. However, evidence supporting the efficacy of immunotherapy for treating AS is limited [37]. Consequently, the effectiveness of the cadonilimab/anlotinib regimen in addressing AS has yet to be thoroughly evaluated. In this particular case, we administered cadonilimab alongside anlotinib to successfully manage and improve multiple metastases of cardiac angiosarcoma (CA), drawing on next-generation sequencing (NGS) findings. To the best of our knowledge, this represents the first case where multiple metastases of CA have been effectively managed by immunotherapy, specifically using the cadonilimab and anlotinib combination. We hope that this unique case can provide valuable insights for the management of similar pathological conditions.

In summary, we describe a 52-year-old woman with left atrial angiosarcoma who developed multiple metastases after surgical resection. The disease was significantly controlled after switching from sintilimab combined with anlotinib to cadonilimab combined with anlotinib. It is important to note that primary cardiac angiosarcoma (CA) is a rare and lethal condition with limited effective treatment options beyond surgery. Immunotherapy, specifically multi-targeted immunotherapy, has provided insights into possible therapeutic approaches for this disease.

This report presents a rare case of left atrial angiosarcoma with metastases in the lung, femoral soft tissue, and other locations. The combination of cadonilimab and anlotinib effectively controlled disease progression. Further research is necessary to validate the efficacy of this treatment approach.

## Data Availability

The data are available from the corresponding author on reasonable request.
